# An Unusual Presentation of Monofocal Methicillin-Resistant Staphylococcus aureus Hepatic Abscess in an Otherwise Healthy Man

**DOI:** 10.7759/cureus.65542

**Published:** 2024-07-27

**Authors:** Ermal Hasalliu, Rita Rehana, Majid Hanna, Mohammed Barawi

**Affiliations:** 1 Internal Medicine, Ascension Macomb Oakland Hospital, Warren, USA; 2 Gastroenterology, Ascension Macomb Oakland Hospital, Warren, USA; 3 Gastroenterology, Ascension Saint John Hospital, Detroit, USA

**Keywords:** liver abscess aspiration, liver abscess drainage, mrsa hepatic abscess, pyogenic hepatic abscess, methicillin resistant staphylococcus aureus (mrsa)

## Abstract

A hepatic abscess is the collection of suppurative matter within the parenchyma of the liver. While most pyogenic liver abscesses (PLAs) are polymicrobial in nature, some rare cases are caused by methicillin-resistant *Staphylococcus aureus* (MRSA). We present a case of a 43 year-old male without evident exposures who presented with abdominal pain and via CT imaging was found to have monofocal MRSA hepatic abscess. An ultrasonography (US)-guided abscess drainage along with a pigtail catheter placement was performed along with antibiotic initiation. This article emphasizes the clinical manifestations of hepatic abscesses and employs literature reviews to offer a comprehensive approach to managing these patient populations.

## Introduction

A hepatic abscess is the collection of suppurative matter within the parenchyma of the liver. The development of an abscess is attributed to hepatic injury or most commonly an intra-abdominal infection disseminated from the portal circulation. Prior studies indicate an increased annual incidence of hepatic abscess in East Asian countries ranging up to 17.6 cases per 100,000 though the incidence of liver abscess in the USA is less common and falls within the range of 3.6 cases per 100,000 people [1,2]. In addition, males are more frequently affected than females, and those within the age group 40-60 years are more vulnerable to developing liver abscesses not attributed to trauma. While most pyogenic liver abscesses (PLAs) are polymicrobial in nature, only 10% of these cases are caused by community acquired *Staphylococcus aureus *(S. aureus) and even fewer by methicillin-resistant S. aureus (MRSA) [3]. We present a case of a 43-year-old male without evident exposures who presented with monofocal MRSA hepatic abscess.

## Case presentation

**Figure 1:**
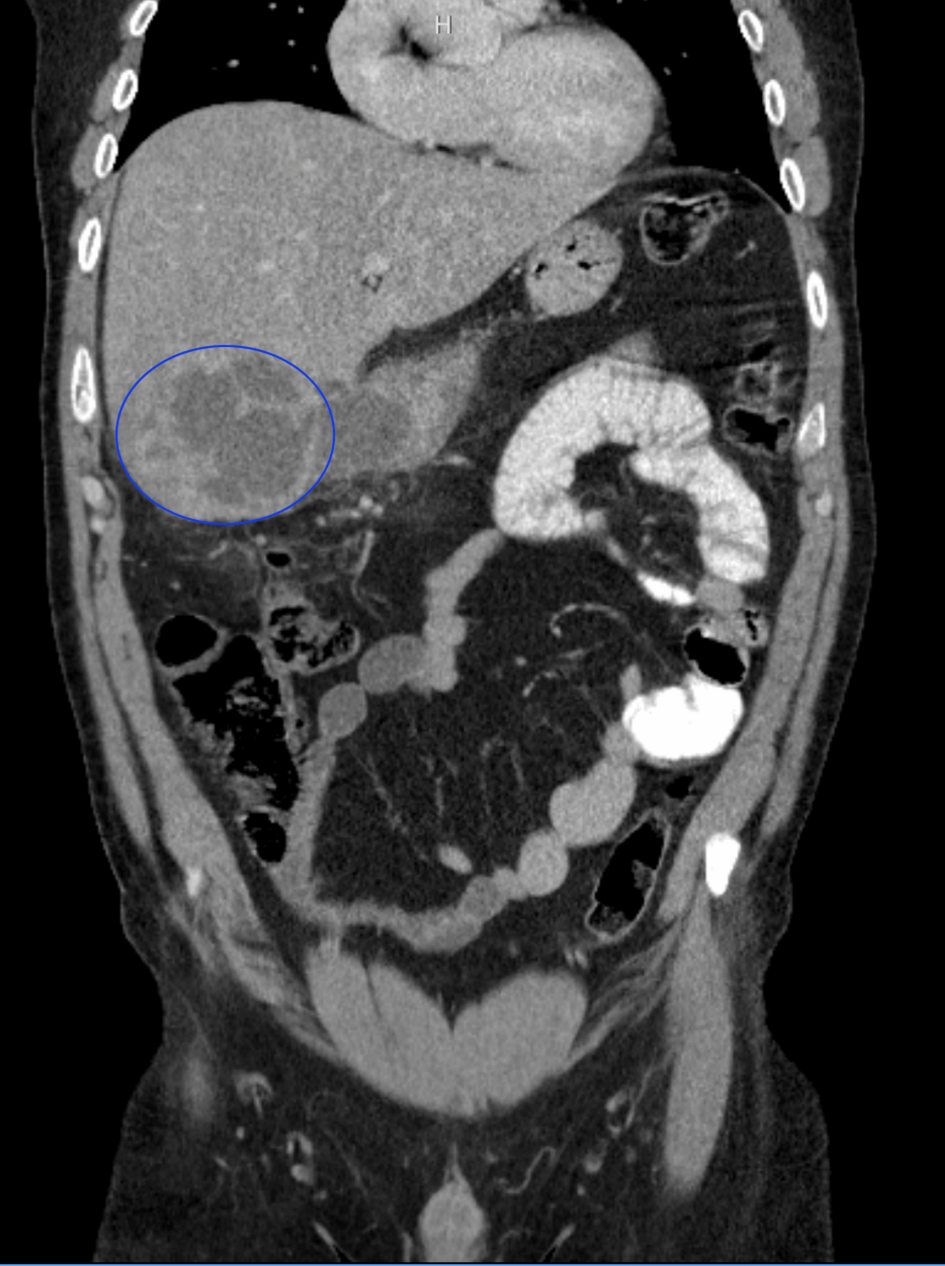
Hepatic abscess found on CT abdomen/pelvis with IV contrast on admission (blue circle)

A 43-year-old male with past medical history of tobacco use disorder presented to the hospital with complaints of a two-month history of abdominal pain associated with subjective fevers and chills. He reported to be in his usual state of health and denied any recent illness, travel, sick contacts, IV drug use, or illicit drug use. The patient denied any nausea, vomiting, dysuria, or changes in bowel habits. He did report occasional alcohol use but denied family history of gastrointestinal-related disorders. Prior to admission, the patient was evaluated by his primary care provider where they ordered a CT abdomen/pelvis with IV contrast which displayed an 8.2 cm x 10.4 cm x 7.2 cm complex cystic lesion in the left liver lobe extending exophytically from the inferior surface of the liver suspicious for abscess (Figure 1), and ultimately he was sent to the hospital for further workup. 

Clinical course

**Figure 2:**
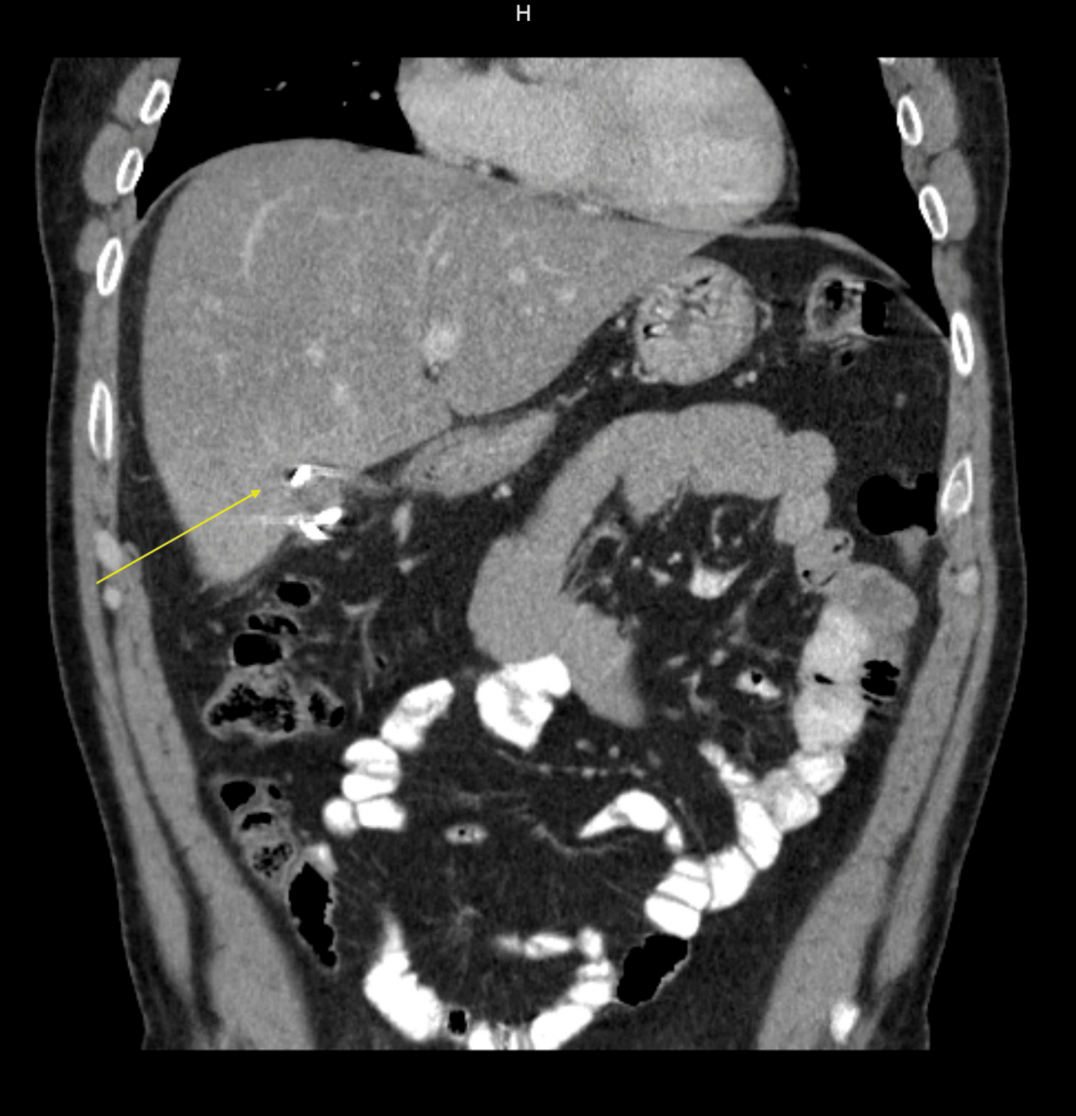
Near complete resolution of hepatic abscess 45 days post intervention (yellow arrow)

In the emergency department, vitals showed a blood pressure of 117/74 mmHg and heart rate of 104 bpm. Pertinent labs were significant for leukocytosis of 16.26 K/mcl, mildly elevated liver enzymes (aspartate transaminase (AST) 46 unit/L and alanine transaminase (ALT) 56 unit/L) and negative hepatitis panel. An abdominal ultrasound demonstrated a complex lesion involving the liver. Ultimately the patient was admitted and evaluated by gastroenterology, infectious disease (ID) and interventional radiology (IR). IR performed an ultrasonography (US)-guided abscess drainage along with a pigtail catheter placement and submitted for cultures while ID initiated Unasyn empirically. Wound cultures returned positive for MRSA while blood cultures remained negative for any growth. Echocardiogram performed during admission did not display any signs of valvular vegetations. Given positive culture results, the patient was discharged on intravenous vancomycin for four weeks. Repeat CT abdomen/pelvis with IV and oral contrast 45 days later ​​displayed near complete resolution of previously seen liver abscess (Figure 2).

## Discussion

The development of PLAs is attributed to a variety of etiologies most notably the microbial invasion of liver parenchyma via hematogenous spread, bacterial dissemination via hepatic artery (disseminated sepsis), ascending cholangitis and necrosis of primary tumor. In rare cases, PLA can arise from trauma, chemo frequency ablation and Caroli disease [4]. As a result of its proximity to the gallbladder, the liver is at its highest risk of bacterial seeding from biliary source most frequently ascending cholangitis, cholecystitis, cholelithiasis and/or perforated gallbladder. Hematogenous spread via portal vein system can lead to liver abscesses as seen in cases of bacteremia arising from underlying abdominal disease, skin infections and inflammatory bowel disease. The common organisms isolated in hepatic abscesses include E. coli (responsible for most cases in the world), Pseudomonas and Klebsiella Pneumonia (K. pneumoniae). While initially predominant in Asia, studies have found that K. pneumoniae has now transcended as the most common isolate in both Asia and the USA and is found in more than 60% of monomicrobial and polymicrobial PLA infections [5]. 

In rare cases, PLA arise from gram positive organisms most notably Streptococcus, Staphylococcus, and anaerobic organisms. Only 10% of these cases are caused by community acquired S. aureus and even fewer by MRSA as seen in our patient. Literature review studies performed by Sofanit et. al alluded to immunocompromised state from chronic disease, skin infection, and incarceration as predisposing risk factors for MRSA liver abscesses while frequent hospitalizations, inflammatory bowel disease and colorectal cancer were attributed to community acquired S. aureus liver abscesses [6]. Other predisposing factors include hepatobiliary surgery and diabetes mellitus [7]. While our patient lacked any of these aforementioned medical conditions, his past alcohol use history could be a potential predisposing factor for developing a liver abscess. Cohort studies performed Wang et al. concluded that incidence of liver abscesses was 3.47-fold greater in the alcohol intoxication cohort than in the non-alcohol intoxication (AI) cohort [8]. 

While cases of PLAs are rare, when present however, they represent a serious risk to patients with mortality rates ranging near 15% prompting expeditious diagnosis and treatment. Clinical manifestation of hepatic abscesses are nonspecific with patients presenting most commonly with fever, chills, abdominal pain, and hepatomegaly. While leukocytosis and anemia followed by increased C-reactive protein (CRP) along with elevated alkaline phosphatase were the most common laboratory findings [9,10]. Due to its nonspecific clinical presentation and laboratory findings, diagnosis of PLAs is highly dependent on clinical suspicion and confirmation with imaging. US and CT imaging are highly diagnostic with tri-phasic enhanced CT providing superior sensitivity. 

Once a PLA is isolated via imaging, drainage, and antibiotic administration are the cornerstone of management. Needle aspiration under the guidance of US or CT is sufficient in small abscesses (<5 cm) though in abscesses larger than 5 cm as seen in our patient, percutaneous drainage with catheter placement reduces recovery time and shortens hospital stay [11]. Surgical drainage may be beneficial in multiple multiloculated abscesses or in case to case basis where percutaneous drainage fails. In patients with a history of biliary procedures or difficult cases, endoscopic retrograde cholangiopancreatography (ERCP) and endoscopic ultrasound (EUS) can be successful in drainage of PLAs [12,13]. Once drainage is performed, wound and blood samples should be sent for culture with empiric antibiotic initiation. Empiric coverage should be tailored to Streptococci, gram negative bacilli, anaerobes, and E. histolytica. Hence, preferred regimen includes piperacillin-tazobactam or third generation cephalosporin with metronidazole.. Antibiotic adjustment is tailored to positive microbiology.

Isolation of bacteria is vital in guiding future management. A case series performed by Qu et. al on Eastern Asian patients found that K. pneumoniae PLA was strongly associated with colorectal cancer, especially those occurring in sigmoid colon and rectum hence necessitating future guidelines [14]. In cases of Streptococcus or Staphylococcus isolated infections, as seen in our patient the focus should be on finding another source of infection. Staphylococcus and Streptococcus infections usually arise from skin infection hence a lack of skin infectious cause creates concern for MRSA bacteremia with hematogenously spread to the liver. The most concerning complication of Staphylococcus/MRSA bacteremia is endocarditis. Hence, blood cultures and echocardiogram are crucial to obtain to rule out valvular vegetations. Fortunately, our patient’s blood culture returned negative for any bacteremia and the echocardiogram did not display any vegetations.

## Conclusions

While hepatic abscesses are rare in nature, when present they are associated with high mortality rate; hence expeditious diagnosis and treatment is vital in survival. The clinical presentation of this condition is often nonspecific, with patients typically experiencing fever, chills, and upper abdominal pain, while laboratory results frequently show leukocytosis, anemia, and elevated CRP levels. Diagnosis relies on imaging (CT or US) while treatment of the condition is catered toward bacterial isolation on culture and appropriate antibiotic coverage. In larger abscesses, needle aspiration and/or percutaneous drainage is vital in reducing recovery time. Microbiology analysis will not only guide management of primary infection but also raise awareness of possible future complications most notably association of K. pneumoniae with colorectal cancer and screening for endocarditis in MRSA abscesses.
